# From Biomechanical Properties to Morphological Variations: Exploring the Interplay between Aortic Valve Cuspidity and Ascending Aortic Aneurysm

**DOI:** 10.3390/jcm13144225

**Published:** 2024-07-19

**Authors:** Ivars Brecs, Sandra Skuja, Vladimir Kasyanov, Valerija Groma, Martins Kalejs, Simons Svirskis, Iveta Ozolanta, Peteris Stradins

**Affiliations:** 1 Faculty of Medicine, Riga Stradins University, 16 Dzirciema Street, LV-1007 Riga, Latvia; vladimirs.kasjanovs@rsu.lv (V.K.); martins.kalejs@stradini.lv (M.K.); iveta.ozolanta@rsu.lv (I.O.); peteris.stradins@stradini.lv (P.S.); 2 Centre of Cardiac Surgery, Pauls Stradins Clinical University Hospital, 13 Pilsonu Street, LV-1002 Riga, Latvia; 3 Joint Laboratory of Electron Microscopy, Riga Stradins University, 9 Kronvalda Boulevard, LV-1010 Riga, Latvia; sandra.skuja@rsu.lv (S.S.); valerija.groma@rsu.lv (V.G.); 4 Laboratory of Biomechanics, Riga Stradins University, 5a Ratsupites Street, LV-1067 Riga, Latvia; 5 Institute of Microbiology and Virology, Riga Stradins University, 5 Ratsupites Street, LV-1067 Riga, Latvia; ssvirskis@latnet.lv

**Keywords:** tricuspid aortic valve, bicuspid aortic valve, ascending thoracic aortic aneurysm, biomechanical properties, morphology

## Abstract

**Background**: This research explores the biomechanical and structural characteristics of ascending thoracic aortic aneurysms (ATAAs), focusing on the differences between bicuspid aortic valve aneurysms (BAV-As) and tricuspid aortic valve aneurysms (TAV-As) with non-dilated aortas to identify specific traits of ATAAs. **Methods**: Clinical characteristics, laboratory indices, and imaging data from 26 adult patients operated on for aneurysms (BAV-A: *n* = 12; TAV-A: *n* = 14) and 13 controls were analyzed. Biomechanical parameters (maximal aortic diameter, strain, and stress) and structural analyses (collagen fiber organization, density, fragmentation, adipocyte deposits, and immune cell infiltration) were assessed. **Results**: Significant differences in biomechanical parameters were observed. Median maximal strain was 40.0% (control), 63.4% (BAV-A), and 45.3% (TAV-A); median maximal stress was 0.59 MPa (control), 0.78 MPa (BAV-A), and 0.48 MPa (TAV-A). BAV-A showed higher tangential modulus and smaller diameter, with substantial collagen fragmentation (*p* < 0.001 vs. TAV and controls). TAV-A exhibited increased collagen density (*p* = 0.025), thickening between media and adventitia layers, and disorganized fibers (*p* = 0.036). BAV-A patients had elevated adipocyte deposits and immune cell infiltration. **Conclusions**: This study highlights distinct pathological profiles associated with different valve anatomies. BAV-A is characterized by smaller diameters, higher biomechanical stress, and significant collagen deterioration, underscoring the necessity for tailored clinical strategies for effective management of thoracic aortic aneurysm.

## 1. Introduction

Between 1990 and 2019, the percentage of deaths (82.1%) and disability-adjusted life years (DALYs) (67%) attributable to thoracic aortic aneurysms (TAAs) rose [1,2]. The ascending aorta (AscAo) can be generally indolent and asymptomatic until reaching a significant size or lead to complications such as aortic dissection or rupture [3]. The prevalence of ascending thoracic aortic aneurysm (ATAA) can vary based on factors such as age, gender, genetics, and underlying health conditions [4,5,6].

An ATAA is primarily characterized by the aorta’s dilatation or expansion [7]. There are several potential risk factors of this, such as bicuspid aortic valve (BAV), connective tissue diseases, hereditary susceptibility, and others [8]. Inevitable changes are also associated with natural aging, leading to alterations in the morphology of the aorta, including changes in luminal diameter, whole length, thickness, and structural components, which have been shown to play a significant role in the pathogenesis of aortic degeneration and the weakening of the aortic tissue [9].

In order to guarantee optimal hemodynamics, the morphology, structural composition, and mechanical characteristics of the aortic wall differ along its descending and ascending segments [10,11]. In addition, the microarchitecture of a healthy human aorta is highly organized in concentric structural units to serve a high degree of flexibility since the aorta is in charge of carrying blood from the heart to the systemic circulation [12,13,14]. However, histological findings reflect degradation of extracellular matrix components (ECMs), which is usually accompanied by calcification in various aortic diseases [13]. In aneurysms, changes in the cellular level include the phenotypic switching of smooth muscle cells and fragmentation and/or loss of fenestrated elastic lamellae, as well as the development of fibrosis [15]. A loss of structural integrity is accompanied by biomechanical changes in the wall of the AscAo [16,17]. 

The structural remodeling is thought to differ in all three aortic wall sublayers: tunica intima (intima, TI), tunica media (media, TM), and tunica adventitia (adventitia, TA) [18,19]. Examining the thickness of elastic lamellae and collagen fibers, along with assessing possible fragmentation and loss of parallel organization and density, provides insight into medical phenomena like aortic dilation progression. However, our understanding as to what determines the site of aneurysm formation remains incomplete [20,21,22,23].

BAV is the most common congenital cardiac abnormality [24]. Controversial data on BAV, accompanied by an increased risk of aneurysm, are reported [25,26,27]. Nevertheless, an examination at the ultrastructural level of dilated BAVs in contrast to non-dilated BAVs indicates a connection with alterations in the mechanical characteristics of the AscAo and an impaired collagen synthesis process implicated in the development of aneurysms [28].

Here, we explore the biomechanical and structural differences between bicuspid aortic valve aneurysm (BAV-A) and tricuspid aortic valve aneurysm (TAV-A), compared with non-dilated aorta.

## 2. Materials and Methods

### 2.1. Subject Grouping Principles, Imaging Technologies, and Aortic Measurements

A cohort of twenty-six adult patients with ATAAs and thirteen controls were included in this study. The patients underwent surgery at Pauls Stradins Clinical University Hospital in Riga, Latvia. Indications for cardiac surgery were based on the 2014 ESC Guidelines on the diagnosis and treatment of aortic diseases and the 2018 EACTS/ESVS Consensus Statement on Treatment of Thoracic Aortic Pathologies. Specimens of the AscAo were collected from each patient according to the planned volume of each operation and were then processed for biomechanical testing and morphological analysis. Tissue samples of the AscAo obtained during autopsies from thirteen individuals with TAV and without a history of previous cardiac surgery were used as controls. All autopsies were performed within 72 h of death. The patients enrolled in this study, who required aneurysm surgery, were further stratified into two groups: (i) twelve patients with BAV-A (BAV group), and (ii) fourteen patients with TAV-A (TAV group). Patients with BAV-A were identified intraoperatively, via presurgical transthoracic echocardiography (TTE) or computed tomography angiography (CTA) imaging. Patients with TAV-A were defined as idiopathic if they had a TAV with no clinical characteristics, family history, or signs of connective tissue disorders.

Calculations for the aortic height index (AHI), length height index (LHI), diameter height index (DHI), and aortic size index (ASI) were performed using the following formulas: AHI = (maximum AscAo diameter cm + AscAo length cm)/patient height m; LHI = AscAo length cm/patient height m; DHI = maximum AscAo diameter cm/patient height m; and ASI = maximum AscAo diameter cm/BSA m^2^ [29,30]. 

This study was approved by the Ethics Committee of Riga Stradins University (Decision No. 4/28.06.2018) and conducted according to the Declaration of Helsinki. Informed consent was obtained from all study participants. Other medical data, such as patients’ medical history, laboratory analyses, and clinical imaging data, were obtained from medical records. 

### 2.2. Biomechanical Tests

Prior to experimentation, the materials were kept in frozen isotonic physiological saline at a temperature of −20 ± 1 °C. Studies on soft biological tissues like arteries and heart valves, which had been frozen and stored under low temperatures, have demonstrated that such storage conditions do not affect the biomechanical characteristics of the materials [31,32,33].

AscAo samples were prepared using a specialized stamp featuring two parallel razor blades. Each specimen was taken from the anterior region of the AscAo and cut circumferentially. These samples were 30–35 mm in length and 5 mm in width. Each specimen was meticulously cleaned, preserving the intima and adventitia layers. Subsequently, the specimens underwent uniaxial tensile testing using a Zwick/Roell (Borken, Germany) BDO-FB0.5TS tensile test machine, equipped with a 50.0 ± 0.1 N load cell and controlled by testXpert II software (Zwick/Roell, Borken, Germany) for data processing. Prior to testing, the thickness of the samples was measured using an optical cathetometer MK-6 (LOMO, Saint Petersburg, Russia) with an accuracy of ±0.01 mm. The samples were stretched at a rate of 5 mm/min until rupture occurred, with all tests conducted at room temperature (21 ± 1 °C). Throughout the experiment, the specimens were kept moist by spraying isotonic physiological solution to prevent tissue dehydration. The collected experimental data were analyzed using testXpert II software to determine maximal stress, maximal strain, and the tangential modulus of elasticity in the linear segment of the stress–strain curve.

The tangential modulus of elasticity (E) describes the rigidity of the tissue. A higher value of this modulus indicates greater stiffness of the tissue. It is calculated as the tangent of the angle formed between the strain axis and the tangential line on the linear segment of the stress–strain curve [34].

The strain of the samples was determined using the formula ε = [(l − l_0_)/l_0_] × 100%, where l represents the deformed length of the sample, and l_0_ denotes the original length of the sample. 

The stress was calculated as σ = F/A, where F stands for the axial force applied to the sample, and A represents the actual cross-sectional area of the sample, calculated under the assumption that the tissue is incompressible.

The incompressibility of the aortic wall material due to the high liquid content was established in [35] and confirmed in [36]. This implies that the volume of the sample remains unchanged during deformation, equivalent to its volume in its original, undeformed state. Consequently, the actual cross-sectional area of the sample is computed as A = A0/(1 + ε), where A0 represents the original cross-sectional area of the sample.

### 2.3. Histopathological Examination

Formalin-fixed, paraffin-embedded tissue sections were stained with a routine hematoxylin and eosin (H&E) technique. After conventional evaluation of samples, both the Picro-Sirius Red Staining kit (Abcam, Cambridge, UK, ab245887) and the Elastica van Gieson staining kit (Merck KGaA (Merck Millipore), Darmstadt, Germany, product no. 1.15974.0002) were used to visualize extracellular matrix elements, following the manufacturer’s protocols. The Picro-Sirius Red-stained sections were additionally examined under interference and polarized light, enhancing the visualization and evaluation of collagen fibers with a Biolar microscope (BIOLAR, Warsaw, Poland) [37]. We assessed fenestrated elastic lamellae and collagen fibers in the media and analyzed collagen fibers in both the intima and adventitia. Both collagen fibers and elastic lamellae were analyzed semi-quantitatively by grading as follows: for relative density (“1”—1–30% of the field of view was covered by collagen fibers/elastic lamellae; “2”—31–60% of the field of view was covered by collagen fibers/elastic lamellae; “3”—61–100% of the field of view was covered by collagen fibers/ elastic lamellae), for thickness (“1”—<50% thick fibers/lamellae; “2”—both tiny and thick fibers /lamellae; “3”—>50% thick fibers/lamellae), for organization (“1”—1–30% parallel alignment to the neighboring fibers/lamellae; “2”—31–60% parallel alignment to the neighboring fibers/lamellae; “3”—61–100% parallel alignment to the neighboring fibers/lamellae), for fragmentation (“1”—<50% fragmented pattern of fibers/lamellae; “2”—fragmented and regular pattern of fibers/lamellae; “3”—>50% fragmented fibers/lamellae), and for inter-lamellar space width (“1”—<50% of the field of view thick inter-lamellar spaces; “2”—distribution of thick and thin inter-lamellar spaces is equal; “3”—>50% of the field of view thick inter-lamellar spaces). Additionally, collagen fibers were assessed by Masson’s trichrome staining (Sigma Aldrich, St. Louis, MO, USA, product no. HT15). The presence of adipocytes and inflammatory infiltrates in the adventitial layer was assessed per visual field using grades on a scale from “1” to “3”.

Morphology of aortic wall tissue samples was analyzed by two independent observers using a light microscope Leica (LEICA, LEITZ DMRB, Wetzlar, Germany) in 10 randomly selected visual fields per each sublayer. Sections were scanned and images were captured using Glissando Slide Scanner (Objective Imaging Ltd., Cambridge, UK).

### 2.4. Scanning Electron Microscopic Examination

For SEM examination, tissue samples from BAV-As and TAV-As were dehydrated using a series of graded acetone solutions (70%, 80%, and 90% acetone in water, and 100% acetone). After that, they were dried using liquid CO_2_ using the critical point method (drying apparatus E3000, Agar Scientific, Stansted, UK). Following the application of a gold coating (automatic sputter coater, JEOL JFC-1300, Akishima, Tokyo, Japan), samples were analyzed at a magnification of 5000–10,000× and an accelerating voltage of 25 kV using the JSM-6490LV (JEOL, Akishima, Tokyo, Japan).

### 2.5. Statistical Data Analysis

Calculations and analyses of biomechanical and morphological data and plotting were performed using GraphPad Prism 9.0 for MacOS (GraphPad Software, La Jolla, CA, USA), Jamovi (version 2.3.11, The Jamovi Project, Sydney, Australia), and JMP 17 (SAS, Cary, NC, USA). Data normality was checked by D’Agostino and Pearson, Anderson–Darling, and Shapiro–Wilk tests, and homogeneity by Brown–Forsyth and Bartlett tests. To compare two groups, when appropriate, the Student’s *t*-test (St) was used; otherwise, the nonparametric Mann–Whitney U-test (MW) was applied. Multigroup comparisons were performed using the nonparametric Kruskal–Wallis (KW) test with the Dwass–Steel–Critchlow–Fligner (DSCF) procedure as a post-hoc analysis. Pairwise comparisons of the numeric ordinal type data were performed by applying a Kruskal–Wallis test with chi-square approximation.

To obtain a more objective overall understanding of the relevant morphological characteristics and aspects, the numerical values of their semi-quantitative assessment were summed up and presented in graphs as scores. In most cases, the median (Md) with an interquartile range (IQR) was used to characterize the central tendency and dispersion of the variables. The respective numbers in the graphs reflect exact *p*-values, and *p*-values ˂ 0.05 were assumed to be statistically significant. For the comparison of the proportions of respective cases in a categorical classification between the groups, the chi-square test with Yates correction (Chi^2^Y) was applied. An unsupervised machine learning approach based on hierarchical cluster analysis was used as a model to identify groups of studied factors.

## 3. Results

### 3.1. Patients’ Characteristics, Diagnostic Imaging Results, and Laboratory Indices Results for Bicuspid and Tricuspid Valve Ascending Aortic Aneurysms

Among the 26 patients with ATAAs included in this study, 12 (46%) had a BAV, and 14 (54%) had a TAV, both confirmed intraoperatively and via presurgical transthoracic echocardiography or computed tomography angiography imaging. There was a statistically significant difference in age between the groups (*p* = 0.0101). Patients in the BAV group were younger, with a median age of 59.50 (IQR 51.12–66.25), compared to the TAV group with a median age of 68.25 (IQR 52.25–74.00). There was no difference between the sexes in the groups. We observed that patients in the BAV group were taller compared to those in the TAV group, with a statistically significant difference (*p* = 0.0382). The mean height of patients in the BAV group was 1.79 m (SD = 0.10), while in the TAV group it was 1.71 m (SD = 0.09). No other anthropometric differences were found.

Echocardiography data revealed severe aortic valve stenosis in seven (58.3%) patients in the BAV group, whereas no patients in the TAV group exhibited severe aortic valve stenosis, resulting in a statistically significant difference between the groups (*p* = 0.0037). Severe aortic valve regurgitation was observed in three (25%) patients with BAV and eight (57.1%) patients with TAV, although this disparity was not statistically significant. Left ventricular ejection fraction (EF) was analyzed, but no statistically significant differences were found. The EF in the BAV group was Md 59% (IQR 55.8–60.0), while in the TAV group, it was Md 50% (IQR 48.3–63.3). In reviewing the CTA data, we observed that the maximum diameter of the AscAo, measured at its widest point, was Md 5.45 cm (IQR 5.33–5.70) in the BAV group, which was statistically significantly higher (*p* = 0.0055) in the TAV group at Md 6.15 cm (IQR 5.60–6.75). The length of the AscAo was also evaluated, measured from the aortic annulus to the origin of the innominate artery. The mean AscAo length was 12.23 cm (SD 1.23) in the BAV group and 11.71 cm (SD 1.45) in the TAV group, with no statistically significant difference between the groups. No differences were found in the dilatation of the aortic root.

When analyzing laboratory indices, it was found that only high-density lipoprotein was statistically significantly higher (*p* = 0.0194) in the TAV group, with an Md 1.99 mmol/L (IQR 1.58–3.80), compared to the BAV group, with an Md 2.43 mmol/L (IQR 2.29–3.51). However, no differences were detected when comparing triglycerides, total cholesterol, and low-density lipoprotein between the groups. 

There were no notable differences observed in the presence of hypertension, diabetes mellitus, significant coronary artery disease, arrhythmias, or smoking among the groups before comprehensive statistical analysis was performed. The patients’ characteristics observed in BAV-A and TAV-A groups are summarized in Table 1.

### 3.2. Biomechanical Analysis of Aortic Wall

**Figure 1:**
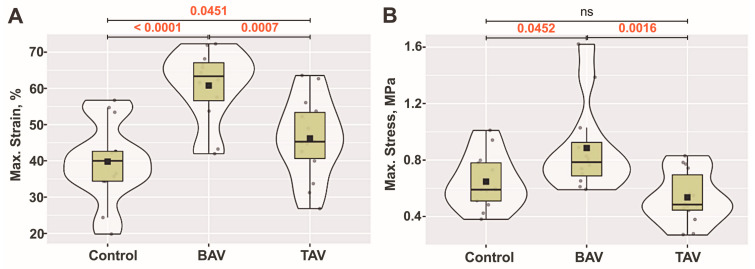
**Reflection of maximal strain and stress in the AscAo wall:** (**A**) Maximal strain (%) for the samples in all groups. (**B**) Maximal stress (MPa) for the samples in all groups. The violin plot represents the shape of the variance of the variable, the box plot shows the median with IQR, and the black square represents the arithmetic mean. Kruskal–Wallis (KW) statistic: (**A**) Chi^2^ = 15.55, *p*_KW_ < 0.001; (**B**) Chi^2^ = 11.43, *p*_KW_ = 0.003; numbers in red indicate significant *p*-values (*p*_DSCF_) of Dwass–Steel–Critchlow–Fligner pairwise comparisons as a post-hoc test.

We observed differences in the maximal strain of the samples among all three groups. The median maximal strain of the samples was Md = 40.0% (IQR 34.3–48.0) in the control group, Md = 63.4% (IQR 54.7–67.8) in the BAV group, and Md = 45.3% (IQR 38.4–54.3) in the TAV group. A statistically significant difference was found between controls and patients with BAVs *(p* < 0.0001), as well as between patients with TAVs and those with BAVs (*p* = 0.0007), and between controls and patients with TAVs (*p* = 0.0451) (Figure 1A).

Upon assessing the maximal stress of the samples, we also found differences. The median maximal stress of the samples was Md = 0.59 MPa (IQR 0.49–0.79) in the control group, Md = 0.78 MPa (IQR 0.66–0.99) in the BAV group, and Md = 0.48 MPa (IQR 0.42–0.75) in the TAV group. We observed a statistically significant difference in the maximal stress of the samples between controls and patients with BAVs (*p* = 0.0452), as well as between patients with TAVs and those with BAVs (*p* = 0.0016). However, there were no statistically significant differences in the maximal stress of the samples between controls and patients with TAVs (Figure 1B).

The highest tangential modulus of elasticity was observed in the BAV group (Md = 3.60 MPa, IQR = 2.78–4.04), followed by the control group (Md = 2.88 MPa, IQR = 2.40–4.47), and the TAV group (Md = 2.67 MPa, IQR = 2.05–3.79).

### 3.3. Structural Analysis of the Aortic Wall Layers

#### 3.3.1. Analysis of Collagenous Content in the Wall of Ascending Aorta 

**Figure 2:**
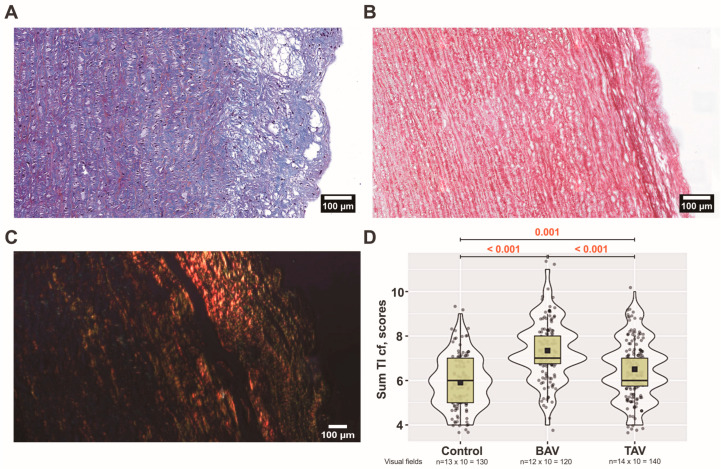
**Arrangement of collagen fibers in the tunica intima (TI):** (**A**) bicuspid aortic valve (BAV) patients’ aortic wall visualized by Massons’s staining procedure (original magnification × 200, scale bar 100 µm); (**B**) BAV patients’ aortic wall visualized by Picro-Sirius Red staining procedure. Note the thickening of collagen between TI and tunica media (TM) (original magnification × 200, scale bar 100 µm). (**C**) In the BAV patient’s TI, under polarized light, Sirius Red-bound collagen fibers appear as bright red structures, creating a stark contrast with the rest of the tissue, which appears dark (original magnification × 100, scale bar 100 µm). (**D**) Scores of all four analyzed parameters of collagen fibers (cf) in the TI layer including all (*n* × 10) visual fields for control, BAV, and tricuspid aortic valve (TAV) patient groups. The violin plot represents the shape of the variance in the variable, the box plot shows the median with the interquartile range (IQR), and the black square represents the arithmetic mean. Kruskal–Wallis (KW) statistic Chi^2^ = 62.8, *p*_KW_ < 0.001; numbers in red indicate significant *p*-values (*p*_DSCF_) of Dwass–Steel–Critchlow–Fligner pairwise comparisons as a post-hoc test.

When comparing three groups, there was a statistically significant difference in the organization of collagen fibers in the intima layer (*p* ˂ 0.005). More organized collagen fibers were observed in controls compared to patients with TAVs (*p* = 0.005) and BAVs (*p* ˂ 0.001). The most fragmented collagen fibers were in BAV group in comparison with TAV group (*p* ˂ 0.001) and controls (*p* ˂ 0.001). The thickness of fibers increased statistically significantly when controls were compared to BAV and TAV (*p* ˂ 0.001). There were no differences in the thickness of collagen fibers between BAV and TAV patients (*p* = 0.107). Also, there were no differences in the collagen density between all three groups in the intima layer. Overall, we observed fewer collagen fibers in the intima layer compared to the other two layers. In intima layer, they are thinner and more chaotic, but not necessarily fragmented compared to other layers. In BAV patients compared to TAV, we observed a thickening of the intima layer much more often (Figure 2A). At the same time, we observed loci with relatively few ECM structural elements. Interestingly, we observed the thickening of collagen fibers between the intima and media layers more often in BAV patients than in TAV patients (Figure 2B,C). Analyzing the sum of all parameters, statistically significant differences were found between all three groups, depicting pronounced changes, especially in the BAV group (Figure 2D).

**Figure 3:**
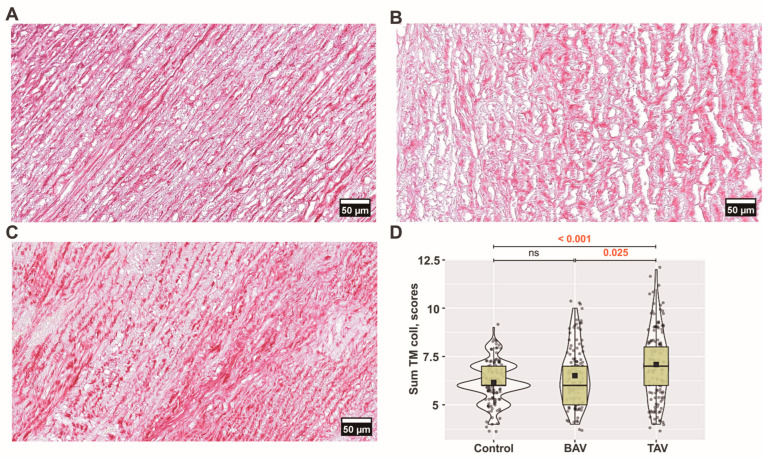
**Arrangement of collagen fibers in the tunica media (TM):** (**A**) controls’; (**B**) bicuspid aortic valve (BAV) patients’; and (**C**) tricuspid aortic valve (TAV) patients’ ascending aortic walls visualized by Picro-Sirius Red staining procedure (original magnification × 400, scale bar 50 µm). (**D**) Scores of all four analyzed parameters of collagen fibers (cf) in the TM layer including all (*n* × 10) visual fields for each group. The violin plot represents the shape of the variance in the variable, the box plot shows the median with the interquartile range (IQR), and the black square represents the arithmetic mean. Kruskal–Wallis (KW) statistic Chi^2^ = 20.0, *p*_KW_ < 0.001; numbers in red indicate significant *p*-values (*p*_DSCF_) of Dwass–Steel–Critchlow–Fligner pairwise comparisons as a post-hoc test.

**Figure 4:**
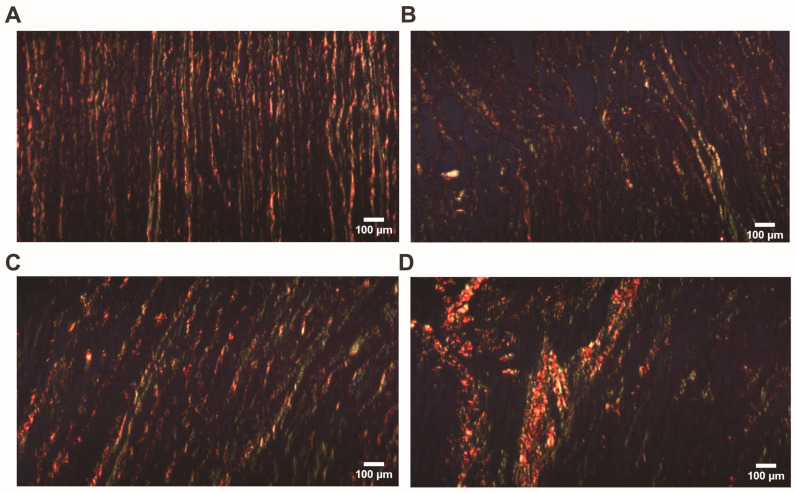
**Arrangement of collagen fibers in the tunica media (TM):** (**A**) controls’; (**B**) bicuspid aortic valve (BAV) patients’; and (**C**,**D**) tricuspid aortic valve (TAV) patients’ ascending aortic walls visualized by Picro-Sirius Red polarization (original magnification × 100, scale bar 100 µm).

We observed a relatively regular arrangement of collagen fibers in the medial layer of the control group (Figure 3A and Figure 4A).

More pronounced collagen fiber fragmentation (*p* = 0.004) and a decrease in collagen bundle density (*p* = 0.047) were observed in the BAV group compared to controls, while in the TAV group, there was a more significant fragmentation compared to controls (*p* = 0.009) (Figure 3B,C and Figure 4B–D). While there were no differences in the thickness of collagen fibers between groups, we found the most significant differences in the organization of collagen fibers when comparing controls with BAV (*p* = 0.017) and TAV (*p* ˂ 0.001) as well as between BAV and TAV (*p* = 0.036) patients.

Comparing TAV and BAV patients, a more pronounced density (*p* = 0.025) was observed in the TAV group, coupled with a more chaotic organization of collagen fibers (*p* = 0.036). Overall, analyzing the sum of all parameters, statistically significant differences were found in the TAV group compared with BAV patients and controls (Figure 3D).

**Figure 5:**
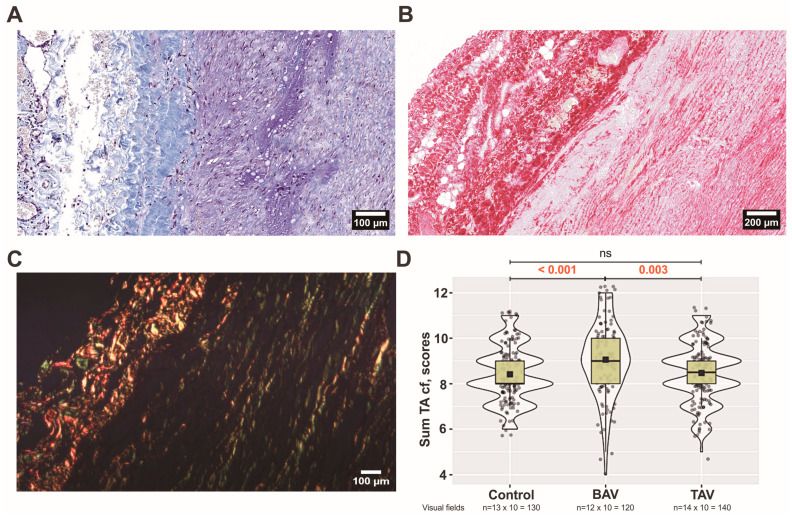
**Arrangement of collagen fibers in the tunica adventitia (TA):** (**A**) tricuspid aortic valve (TAV) patients’ aortic wall visualized by Massons’s staining procedure (original magnification × 200, scale bar 100 µm); (**B**) TAV patients’ aortic wall visualized by Picro-Sirius Red staining procedure. Note the thickening of collagen between tunica media and TA (original magnification × 100, scale bar 200 µm). (**C**) In the TAV patient’s TA, under polarized light, Sirius Red-bound collagen fibers appear as bright red structures, creating a stark contrast with the rest of the tissue, which appears dark (original magnification × 100, scale bar 100 µm). (**D**) Scores of all four analyzed parameters of collagen fibers (cf) in the TA layer including all (*n* × 10) visual fields for each group. The violin plot represents the shape of the variance in the variable, the box plot shows the median with the interquartile range (IQR), and the black square represents the arithmetic mean. Kruskal–Wallis (KW) statistic Chi^2^ = 16.2, *p*_KW_ < 0.001; numbers in red indicate significant *p*-values (*p*_DSCF_) of Dwass–Steel–Critchlow–Fligner pairwise comparisons as a post-hoc test.

Both the relative density and fragmentation of bundles of collagen fibers increased in the BAV group compared with TAV patients and controls in the adventitia layer (*p* < 0.001 and *p* < 0.001, *p* < 0.001, and *p* = 0.004, respectively). There were no differences between groups in the analysis of other parameters. In contrast to BAV patients, in the TAV group, we observed a marked thickening of collagen fibers between the medial and adventitial layers (Figure 5A). In this case, the medial region showing thickening often had a reduced quantity of ECM elements (Figure 5B). In addition, we found that collagen fibers had lost their regular arrangement and often demonstrated a chaotic orientation (Figure 5C). Analyzing the sum of all parameters, statistically significant differences were found in the BAV group compared with TAV patients and controls (Figure 5D). 

**Figure 6:**
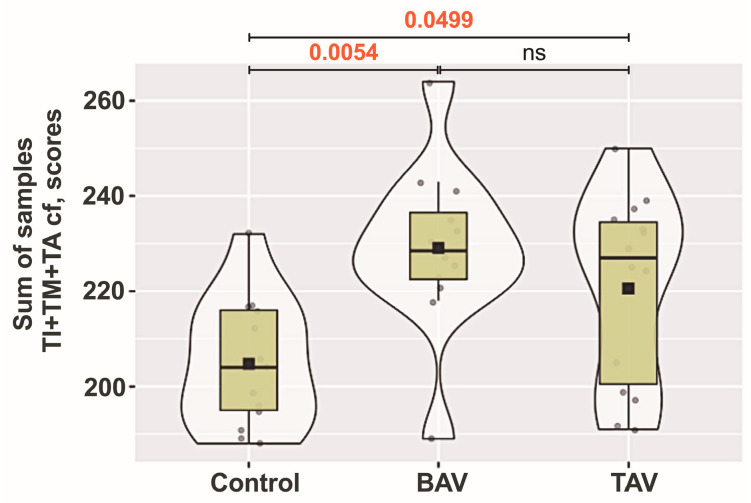
**Analysis of collagen fiber (cf) parameters across aortic wall layers:** tunica intima (TI), tunica media (TM), and tunica adventitia (TA) layers of controls, and bicuspid aortic valve (BAV) and tricuspid aortic valve (TAV) patient groups. The violin plot represents the shape of the variance in the variable, the box plot shows the median with the interquartile range (IQR), and the black square represents the arithmetic mean. Kruskal–Wallis (KW) statistic Chi^2^ = 10.67; *p*_KW_ = 0.005; numbers in red indicate significant *p*-values (*p*_DSCF_) of Dwass–Steel–Critchlow–Fligner pairwise comparisons as a post-hoc test.

Upon analyzing the summarized semiquantitative characteristics of all parameters on collagen fibers in all three aortic wall layers, statistically significant differences were found between controls and both BAV and TAV groups. The pooled total scores for collagen fibers in all three layers of the aorta are shown in Figure 6.

**Figure 7:**
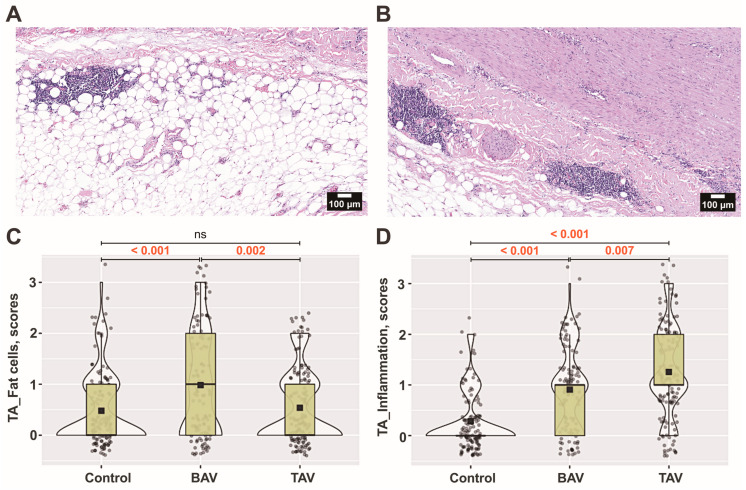
**The microcomposition of the tunica adventitia (TA) layer.** The presence of adipocytes and loci with inflammatory cells in the aortic wall of (**A**) bicuspid aortic valve (BAV) patients and (**B**) tricuspid aortic valve (TAV) patients, as defined by hematoxylin and eosin staining (original magnification × 100, scale bar 100 µm). The numerical values of their semiquantitative assessment presented as scores of (**C**) fat cells and (**D**) inflammation in the TA layer including all (*n* × 10) visual fields for each group. The violin plot represents the shape of the variance in the variable, the box plot shows the median with the interquartile range (IQR), and the black square represents the arithmetic mean. Kruskal–Wallis (KW) statistic (**C**) Chi^2^ = 18.9, *p*_KW_ < 0.001; (**D**) Chi^2^ = 93.1, *p*_KW_ < 0.001; numbers in red indicate significant *p*-values (*p*_DSCF_) of Dwass–Steel–Critchlow–Fligner pairwise comparisons as a post-hoc test.

Besides the changes in density and fragmentation of collagen fibers, there were increased accumulations of adipocytes, especially in the BAV group. Both TAV and BAV patients showed more pronounced adipose cell aggregations (*p* < 0.001) compared to controls (Figure 7A–C).

We found a statistically significant increase in the number of immune cells in both the TAV and BAV groups compared to controls (Figure 7A,B,D). In the adventitia layer of the TAV group, loci with inflammatory cell infiltrates were more common than in the BAV group or controls (*p* = 0.007 and *p* < 0.001, respectively). We observed that an increased number of immune cells in the adventitia often indicates their presence in the media (Figure 7B). 

#### 3.3.2. Analysis of Elastic Membranes in the Medial Layer of Ascending Aorta

**Figure 8:**
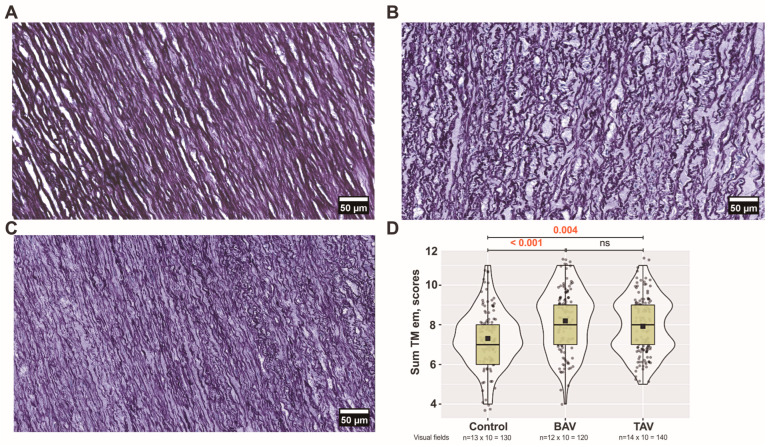
**Arrangement of elastic membranes in the tunica media (TM) layer:** (**A**) controls’; (**B**) bicuspid aortic valve (BAV) patients’; and (**C**) tricuspid aortic valve (TAV) patients’ ascending aortas visualized by van Gieson’s staining (original magnification × 400, scale bar 50 µm). (**D**) sum of all five analyzed parameters of elastic membranes including all (*n* × 10) visual fields for each group. The violin plot represents the shape of the variance in the variable, the box plot shows the median with tricuspid aortic valve (IQR), and the black square represents the arithmetic mean. Kruskal–Wallis (KW) statistic Chi^2^ = 20.8, *p*_KW_ < 0.001; numbers in red indicate significant *p*-values (*p*_DSCF_) of Dwass–Steel–Critchlow–Fligner pairwise comparisons as a post-hoc test.

We found more pronounced changes in all parameters of elastic membranes when the media were analyzed in all three groups. In comparison to both the TAV group and controls, we consistently observed the greatest alterations in the BAV group. Decreased lamellae density and, conversely, increased inter-lamellar spaces were observed in the BAV group compared to the TAV and control groups (*p* < 0.001). Thicker and more disrupted elastic membranes were detected in both the BAV and TAV patient groups compared to the those in control group (Figure 8A–C). The elastic lamellae, which appear as parallel-arranged sheets in controls, had lost their regular arrangement, especially in the media of BAV patients. Overall, analyzing the sum of all parameters, statistically significant differences were found in both BAV and TAV groups compared with controls (Figure 8D). 

### 3.4. Ultrastructural Analysis of the Aortic Wall Using Scanning Electron Microscopy

**Figure 9:**
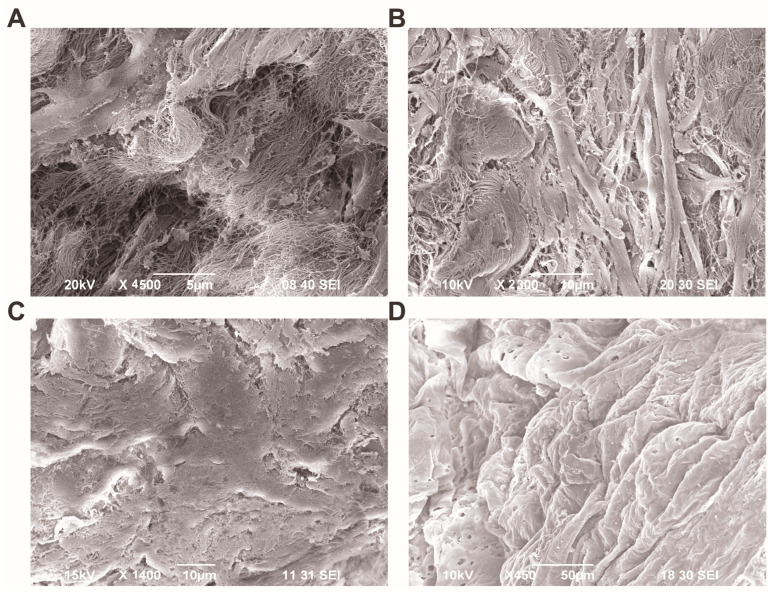
Scanning electron microscopy (SEM) of aortic wall of tricuspid aortic valve (TAV) and bicuspid aortic valve (BAV) patients. (**A**) Representative microphotography depicting the tunica media of TAV patient, scale bar 5 µm; (**B**) a luminal view of the tunica intima showing the endothelial lining of TAV patient, scale bar 10 µm; (**C**) a representative microphotography depicting the tunica media of BAV patient, scale bar 10 µm; (**D**) a luminal view of the tunica intima showing the endothelial lining of BAV patient, scale bar 50 µm.

Under SEM examination, inter-lamellar spaces present between the elastic membranes were observed to be enlarged, and irregularly shaped delaminated spaces were occupied by disrupted collagen fibers, especially in the media of TAV patients (Figure 9A). In BAV patients, changes in the architecture of the medial layer were observed. We found that elastic membranes were weakened and had lost their regular arrangement. These changes were accompanied by an increase in chaotically oriented collagen fibers between them (Figure 9B). 

It is noteworthy that, in addition to the changes observed in the light microscope in the intimal layer in BAV patients, pronounced alterations were observed on the luminal side of the aorta in the scanning electron microscope. The endothelial layer exhibited significant folding; the folds were irregular, and the endothelial cells displayed an increased number of pores compared to those of TAV patients (Figure 9C,D). 

### 3.5. A Comprehensive Analysis of Morphological, Biomechanical, and Clinical Variations Shedding Light on the Unique Characteristics of TAV and BAV Patients

**Figure 10:**
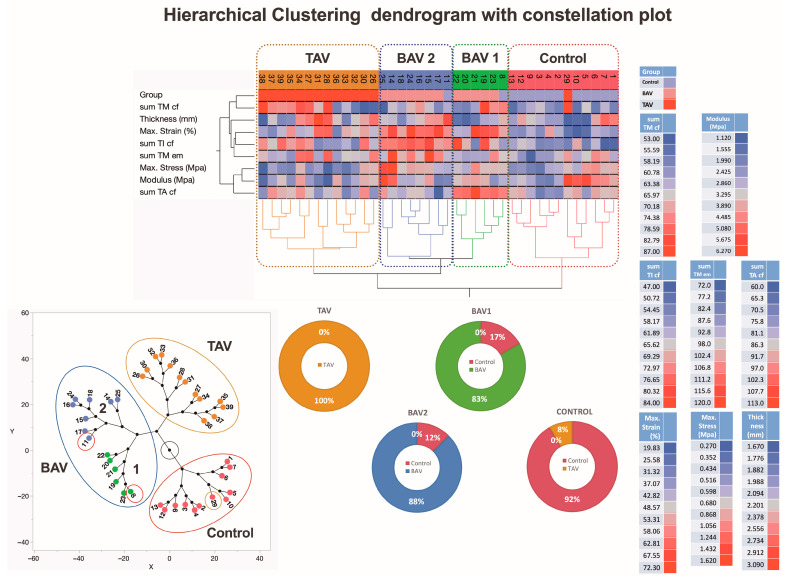
**A dendrogram visually represents hierarchical clustering, illustrating relationships within sets of data.** It comprises stacked branches (clades) that progressively divide into smaller branches. At the lowest level, individual elements are depicted, and as one moves upward, these elements are grouped based on attributes, forming clusters that become progressively fewer. The terminal points of each clade, referred to as leaves, represent the actual data. In this specific context, the data sets encapsulate a sum of parameters analyzed on collagen fibers and elastic membranes in all three layers (TI, TM, and TA) of the aortic wall from 39 individuals, categorized into groups with TAVs, BAVs, and controls. Additionally, details about two biomechanical measurements and thickness data of the ascending aortas (depicted in the right lower part of the figure) are incorporated. Variables are scaled in colors from blue to red, representing values ranging from the lowest to the highest. TAV, BAV, and control specimens are categorized into four subtypes based on differences in histopathology, biomechanical, and clinical measurements. The dendrogram’s constellation plot, located in the left lower part of the figure, emphasizes three major data clusters, each with 1–2 subclusters. The lower middle part of the figure shows the heterogeneity of the BAV and TAV groups. Abbreviations: TI—tunica intima; TM—tunica media; TA—tunica adventitia; cf—collagen fibers; em—elastic membranes.

The hierarchical clustering analysis of the summarized morphological and biomechanical measurement data identified two clusters of patients with BAV, one cluster with TAV, and one cluster of control subjects (Figure 10). Interestingly, the analysis clearly identified two data clusters for the TAV and control groups but divided the BAV data cluster into two separate groups, BAV1 and BAV2. Many of the parameters show higher rates in the blue-colored BAV2 cluster compared to the brown-colored TAV cluster. On the other hand, some individuals from the control group are represented in the conditional BAV1 and BAV2 clusters, one in each. In addition, one representative from the TAV group is identified in one control subcluster due to the similarity of the activity spectrum of the relevant parameters. The proportion of representatives of the control individuals as well as the TAV and BAV patient groups in each of the main clusters is shown in the pie charts.

**Figure 11:**
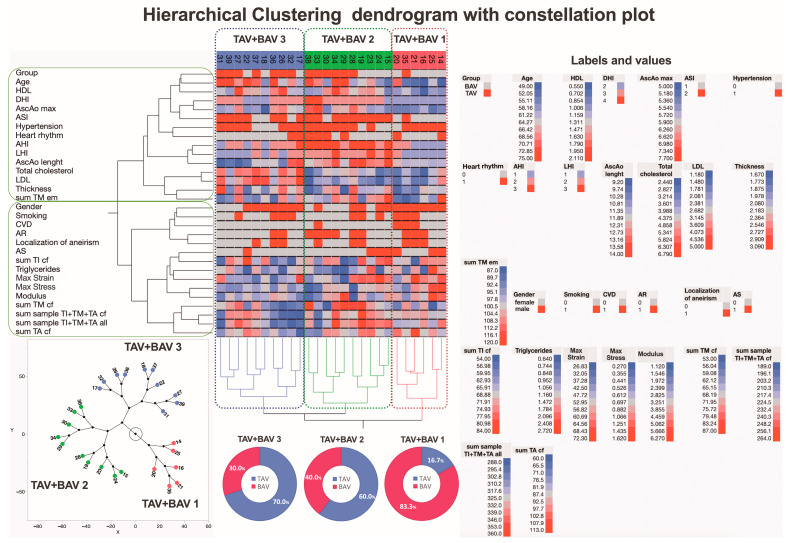
**A dendrogram visually represents hierarchical clustering, illustrating relationships within sets of data.** It comprises stacked branches (clades) that progressively divide into smaller branches. At the lowest level, individual elements are depicted, and as one moves upward, these elements are grouped based on attributes, forming clusters that become progressively fewer. The terminal points of each clade, referred to as leaves, represent the actual data. In this specific context, the data sets encapsulate a sum of parameters analyzed on collagen fibers and elastic membranes in all three layers (TI, TM, and TA) of the aortic wall from 26 patients, categorized into those with BAV and TAV. Additionally, details about two biomechanical measurements and clinical data on the ascending aortas (depicted in the right part of the figure) are incorporated. Variables are scaled in colors from blue to red, representing values ranging from the lowest to the highest. BAV and TAV patients are categorized into three subtypes based on differences in histopathology, biomechanical, and clinical measurements. The dendrogram’s constellation plot, located in the left lower part of the figure, emphasizes three major data clusters with different proportion of TAV and BAV that is shown in the pie charts. Abbreviations: HDL—high-density lipoprotein; DHI—diameter height index; AscAo—ascending aorta; ASI—aortic size index; AHI—aortic height index; LHI—length height index; LDL—low-density lipoprotein; CVD—cardiovascular disease; AR—aortic valve regurgitation; AS—aortic valve stenosis; tunica intima—TI; tunica media—TM; tunica adventitia—TA; cf—collagen fibers; em—elastic membranes.

In this study, hierarchical clustering was used to explore similarities and differences in the summarized morphological evaluation scores, biomechanical measurements, and clinical variations of the aortic valves (Figure 11). Notably, the inclusion of clinical variations in the aortic wall in the analysis identified three data clusters. One cluster matched a predefined group closely, while the other two included parameters from both predefined groups. This underscores the importance of clinical variations, suggesting that the subgroups created may not fully reflect the groups indicating the development of ATAA formation.

**Figure 12:**
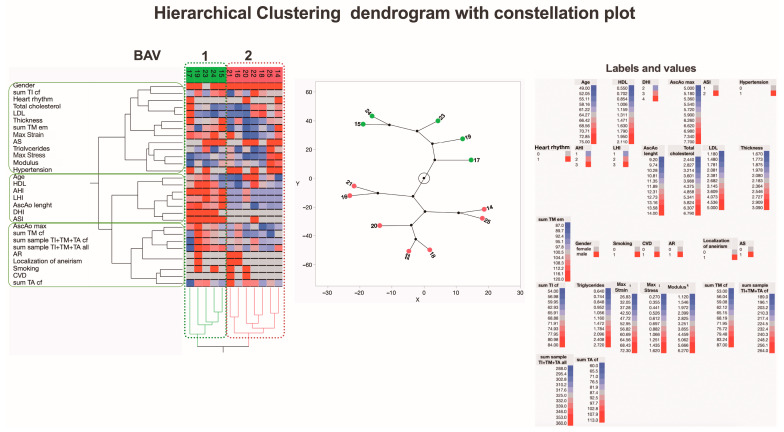
**A dendrogram visually represents hierarchical clustering, illustrating relationships within sets of data.** It comprises stacked branches (clades) that progressively divide into smaller branches. At the lowest level, individual elements are depicted, and as one moves upward, these elements are grouped based on attributes, forming clusters that become progressively fewer. The terminal points of each clade, referred to as leaves, represent the actual data. In this specific context, the data sets encapsulate a sum of parameters analyzed on collagen fibers and elastic membranes in all three layers (TI, TM, and TA) of the aortic wall from 12 patients with BAV. Additionally, details about two biomechanical measurements and clinical data on the ascending aortas (depicted in the right part of the figure) are incorporated. Variables are scaled in colors from blue to red, representing values ranging from the lowest to the highest. BAV patients are categorized into two subtypes based on differences in histopathology, biomechanical, and clinical measurements. The dendrogram’s constellation plot, located in the middle part of the figure, emphasizes two major data clusters. Abbreviations: HDL—high-density lipoprotein; DHI—diameter height index; AscAo—ascending aorta; ASI—aortic size index; AHI—aortic height index; LHI—length height index; LDL—low-density lipoprotein; CVD—cardiovascular disease; AR—aortic valve regurgitation; AS—aortic valve stenosis; TI—tunica intima; TM—tunica media; TA—tunica adventitia; cf—collagen fibers; em—elastic membranes.

Recognizing that the inclusion of clinical parameters in the analysis generates new subgroups, we conducted an in-depth analysis of the BAV and TAV patient groups separately to identify potential distinguishing factors. The first subjects analyzed were BAV patients, categorized into two new subgroups, BAV1 and BAV2, using hierarchical clustering (Figure 12).

**Figure 13:**
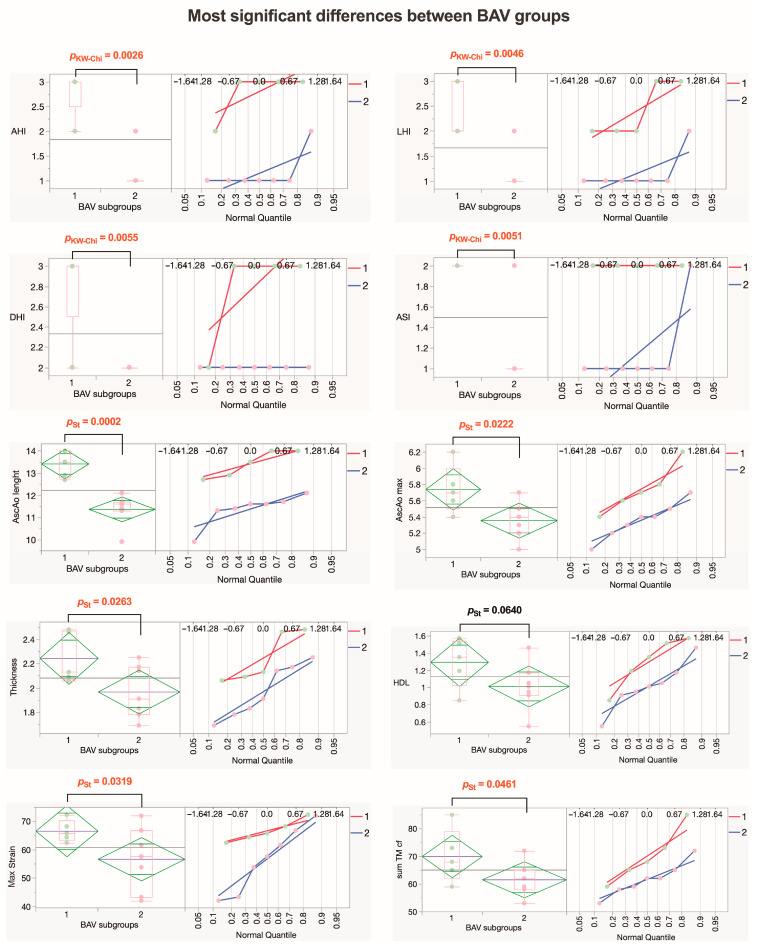
**Parameters that exhibit the most significant differences within the BAV group.** The diamond plot represents group mean of the variable with 95% CI; the box plot shows the median with IQR; numbers in red indicate significant *p*-values (*p*_KW-Chi_—numeric ordinal type data comparison using Kruskal–Wallis test with chi-square approximation; *p*_St_—Student’s *t*-test). Abbreviations: AHI—aortic height index; LHI—length height index; DHI—diameter height index; ASI—aortic size index; AscAo—ascending aorta; HDL—high-density lipoprotein; TM—tunica media; cf—collagen fibers.

Looking for the most significant differences between the BAV1 and BAV2 subgroups, we found that BAV1 had greater values for risk indexes (AHI, LHI, DHI, and ASI) for long-term adverse aortic events (AAEs), with statistically significant differences. The maximal diameter and length of the ascending aorta were also greater in the BAV1 subgroup, and the AscAo wall was thicker in this subgroup, both with statistically significant differences. Interestingly, in the BAV1 subgroup, the maximum strain was greater with a statistically significant difference (*p* = 0.0319), while the summarized morphological evaluation scores for collagen fibers in media tended to have higher values but did not reach statistical significance (*p* = 0.0686). Values for HDL also tended to be higher in the BAV1 subgroup (Figure 13).

**Figure 14:**
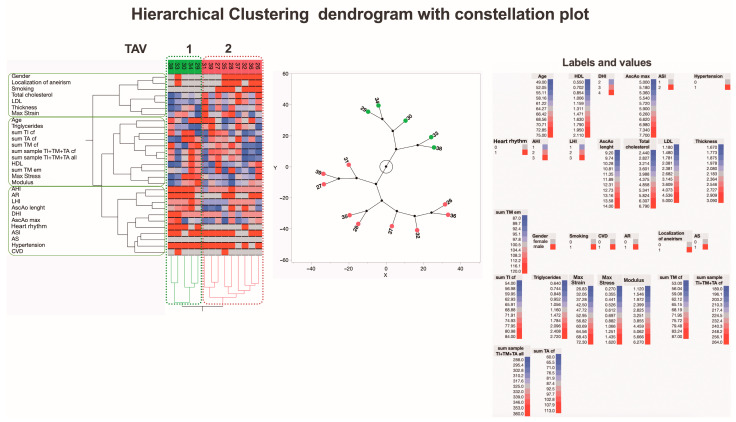
**A dendrogram visually represents hierarchical clustering, illustrating relationships within sets of data.** It comprises stacked branches (clades) that progressively divide into smaller branches. At the lowest level, individual elements are depicted, and as one moves upward, these elements are grouped based on attributes, forming clusters that become progressively fewer. The terminal points of each clade, referred to as leaves, represent the actual data. In this specific context, the data sets encapsulate a sum of parameters analyzed on collagen fibers and elastic membranes in all three layers (TI, TM, and TA) of the aortic wall from 14 patients with TAV. Variables are scaled in colors from blue to red, representing values ranging from the lowest to the highest. TAV patients are categorized into two subtypes based on differences in histopathology, biomechanical, and clinical measurements. The dendrogram’s constellation plot, located in the middle part of the figure, emphasizes two major data clusters. Abbreviations: HDL—high-density lipoprotein; DHI—diameter height index; AscAo—ascending aorta; ASI—aortic size index; AHI—aortic height index; LHI—length height index; LDL—low-density lipoprotein; CVD—cardiovascular disease; AR—aortic valve regurgitation; AS—aortic valve stenosis; TI—tunica intima; TM—tunica media; TA—tunica adventitia; cf—collagen fibers; em—elastic membranes.

**Figure 15:**
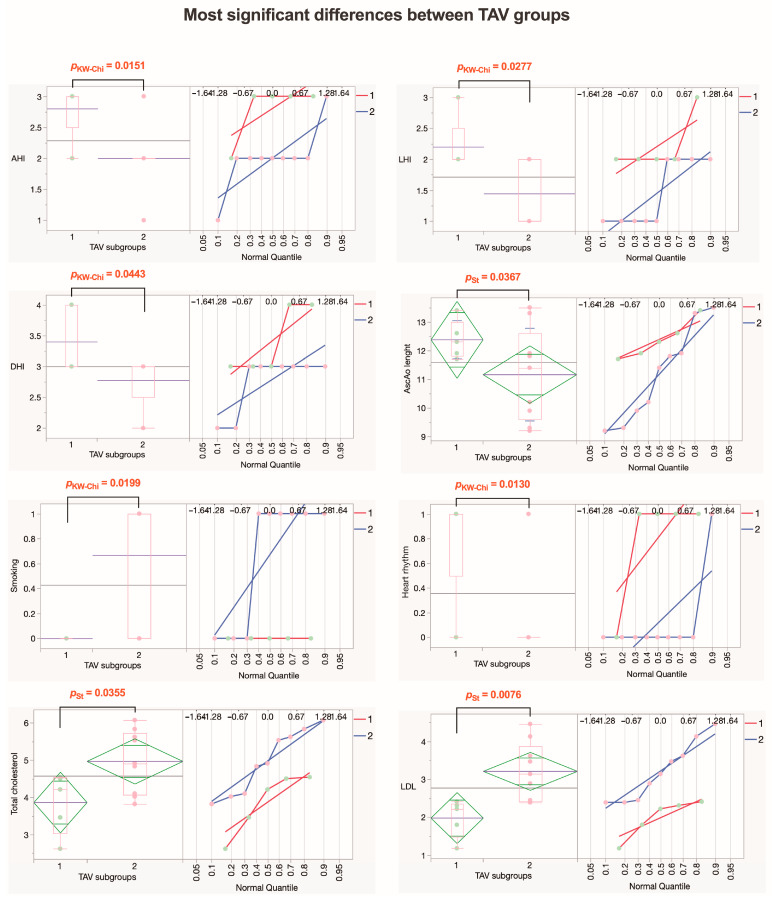
**Parameters that exhibit the most significant differences within the TAV group.** The diamond plot represents group mean of the variable with 95% CI; the box plot shows the median with IQR; numbers in red indicate significant *p*-values (p_KW-Chi_—numeric ordinal type data comparison using Kruskal–Wallis test with chi-square approximation; p_St_—Student’s *t*-test). Abbreviations: AHI—aortic height index; LHI—length height index; DHI—diameter height index; AscAo—ascending aorta; LDL—low-density lipoprotein.

Next, we analyzed TAV patients (Figure 14). Analysis showed that the TAV1 patient subgroup had higher values for AAEs risk indexes AHI and LHI, with statistically significant differences, while DHI tended to be higher in the TAV1 subgroup (*p* = 0.0539). Additionally, the TAV1 subgroup had a greater length of AscAo and higher rates of arrhythmias, both with statistically significant differences. Furthermore, TAV2 patients were more often smokers (*p* = 0.0245) with higher LDL rates (*p* = 0.0076), and rates for total cholesterol tended to be higher (*p* = 0.0830). However, we did not find statistically significant differences between summarized morphological evaluation scores and biomechanical measurements between the TAV1 and TAV2 subgroups (Figure 15).

## 4. Discussion

This research provides a thorough understanding of morphological changes in the ascending aortic wall, laying the groundwork for comparing biomechanical test results and enhancing our comprehension of clinical occurrences in aneurysms in BAV and TAV patients. The detailed analysis reveals ECM structural variations, potentially influencing the interpretation of surgical outcomes and informing future interventions. 

In this study, we found that morphological distinctions impact all three layers of the aortic wall to varying extents in the BAV and TAV patient groups. Initially, we scrutinized diverse parameters related to changes in collagen fibers in each layer, supplementing observations of these parameters with those of elastic lamellae in the middle layer of the aortic wall. The observed morphological changes may explain clinically detected delamination during aortic wall dissection, particularly in BAV patients between the intimal and medial layers and in TAV patients between the medial and adventitial layers. This may indicate and help pinpoint the location of the primary entry tear, but we did not find comparable data or evidence in the literature. When acknowledging the importance of complete and thorough reconstruction of the aortic wall during surgery, especially by accurately localizing the adventitia layer, evidence suggests that the TA, being three times stronger than the other two layers, provides significant durability in the postoperative period [38].

Our biomechanical investigation shows differences in the maximal strain of the samples among all three groups. Statistically significant differences were found between controls and patients with BAV, between patients with TAV and those with BAV, and between controls and patients with TAV. Significant differences in maximal stress were also found between controls and patients with BAV, as well as between patients with TAV and those with BAV. However, there were no statistically significant differences in the maximal stress between controls and patients with TAV (Figure 1). This is due to significant differences in the structural organization and density of collagen fibers in all three aortic wall layers, particularly in BAV-A cases. These results are consistent with studies available in the literature [39,40].

A comparison of maximal strain (Figure 1A) with the sum of the collagen fiber scores in the intima, media, and adventitia showed that higher maximal strain is associated with higher scores in both the BAV and TAV groups, as confirmed morphologically (Figure 6). Simultaneously, lower scores, confirmed by morphological assessment, are associated with lower maximal strain in the control group. In line with our findings, Chung et al. discovered that increased stiffness in the aorta correlated with higher levels of collagen [41]. A comparison of maximal stress (Figure 1B) with the sum of the elastic membrane scores (Figure 8D) showed that in the BAV group, greater maximal stress is associated with higher score, as confirmed morphologically. It is worth noting that decreased density and disrupted elastic membranes were detected more frequently in BAV patients compared to the control group. Furthermore, greater deformation is associated with a higher sum of elastic membrane scores only in the BAV group. Consistent with our findings, Bollache et al. showed that elastic fiber thinning was associated with increased tissue stiffness [42]. Pichamuthu et al. [40] compared aortic wall tensile stress between BAV and TAV ATAAs to determine whether the collagen content of the ATAA wall is associated with tensile strength and valve phenotype. They found that the increased tensile strength in BAV ATAAs, despite uniform collagen content between groups, indicates that microstructural changes in collagen contribute to BAV-associated aortopathy. In turn, the application of hierarchical clustering analysis in this study revealed that BAV patients were split into two subgroups: BAV1 and BAV2. BAV1 exhibited higher risk indexes, aortic wall structural change and thickness scores, and tangential modulus of elasticity compared to both BAV2 and the TAV group, highlighting significant differences in clinical, morphological, and biomechanical parameters. Furthermore, the analysis showed that summarized scores from morphological evaluations and biomechanical measurements may not fully reflect all factors contributing to the development of ATAA formation [43,44]. To summarize our findings on ATAAs, higher maximal strain is associated with higher collagen fiber scores in both BAV and TAV groups, indicating increased stiffness in the aorta with higher collagen levels. In the BAV group, greater maximal stress is linked to higher elastic membrane scores, with decreased density and disrupted elastic membranes more frequently observed compared to the control group.

We found alterations in the intimal layer in both the BAV and TAV groups compared to controls. These changes affect the analyzed parameters of collagen fibers, including organization, fragmentation, and thickness. Iliopoulos et al. found that wall thickness measurements were higher for the intima when comparing ascending thoracic aortic aneurysms to control samples [45]. These findings are in line with our results, as we observed a thickening of the intima in the BAV group. Importantly, we also noted marked irregular folding of the endothelial lining in the BAV group compared to the TAV group, likely contributing to increased measurements of the overall wall thickness (Figure 9C,D). Elastic membranes were weakened and had lost their regular arrangement. The endothelial layer exhibited significant folding; the folds were irregular, and the endothelial cells displayed an increased number of pores compared to those of TAV patients. All of this leads to the fact that the maximal strain and stress for the BAV group are significantly higher than for the TAV group.

Interestingly, Miura explored the aging process of the renal arteries, revealing that the intima compensates for the mechanical weakness of the media, particularly in response to increased blood pressure [46]. He disclosed that as individuals age, renal arteries exhibit an elliptical shape, indicating inward hypertrophy of the intima while concurrently experiencing outward hypertrophy of the media. In the process of aging, the renal arteries lose elasticity, and the media and the internal and external elastic laminae exhibit reduced stiffness. Such structural changes with age undoubtedly lead to significant changes in biomechanical parameters, particularly an increase in maximal strain. We found that in the medial layer, collagen fiber fragmentation and disorganization increased in both BAV and TAV patients compared to controls. Simultaneously, we detected a decrease in both elastic lamellae and collagen fiber density, especially notable in patients from the BAV group. These findings, coupled with the greater strain observed in the BAV group, align with those described by Pichamuthu et al. [40]. Notably, simultaneously, patients with BAVs displayed loosening of elastic membranes, alongside an augmentation in inter-membrane gaps, mirroring the findings reported by Pisano et al. [47]. Our observations support the findings of Karalko’s study, which depicted the accumulation of mucoid ECM between disorganized fibers in the TAV group [48]. 

In our study, the most substantial changes in the adventitial layer were observed in the BAV group, characterized by increased collagen fiber density and greater fragmentation. In the TAV group, we observed pronounced compaction of collagen fibers between the adventitia and media, indicating a more depleted ECM in the media part in such cases. Additionally, we found areas of inflammatory cells in the adventitial layer, especially in patients with TAVs, which aligns with the findings of Rodella et al. and Gross et al. [49,50]. Of note, Doderer et al. reported the presence of large adipocyte clusters in the adventitial layer of the abdominal aorta in aneurysm-stricken patients as a potential contributor to aortic wall weakening [51]. These findings are consistent with our results, as we observed extensive adipocyte aggregation within the adventitial layer, especially in the BAV group. Also, it is worth noting that a recent study suggests adipocytes located in the adventitia may play a protective role in preventing neointimal formation in the femoral arteries of experimental animals [52]. However, our findings within a group of patients with BAVs revealed an increased occurrence of adipocytes in the adventitial layer, accompanied by indications of hyperplasia in the intimal layer. 

Our study presents significant evidence of diversity among patients with BAV and TAV. Combining our results, we observed that the heterogeneity within the BAV group stems from changes in collagen fibers across the entire aortic wall, whereas for TAV patients, heterogeneity is predominantly associated with various clinical factors (Figure 13 and Figure 15). The insights gained from this study suggest several potential interventions. For BAV patients, targeted therapies aimed at stabilizing collagen fibers and elastic membranes may reduce the risk of dissection and improve surgical outcomes. Regular monitoring of aortic wall morphology through advanced imaging techniques could help in early detection of detrimental changes and timely intervention. For TAV patients, addressing the clinical aspects of heterogeneity through personalized treatment plans that focus on inflammation and ECM depletion may enhance patient management. Additionally, understanding the role of adipocytes in the adventitial layer could lead to new therapeutic strategies to prevent aortic wall weakening.

Our findings need to be interpreted considering criticism and limitations. One of the main concerns is the relatively modest sample size; nevertheless, through further stratification, we established nearly equal groups, facilitating statistical analysis and comparisons. Another issue to consider is the assessment approach used in this study for morphological applications and biomechanical testing. The biomechanical features of the aortic wall were estimated by considering the entire thickness of the vessel. A more meticulous approach was used to evaluate the fine morphology of the aortic wall layers separately, with a particular focus on the collagen and elastic membrane content. Currently, few studies have demonstrated the possibility of obtaining detailed biomechanical characteristics by subdividing the aortic wall into its individual layers. Future studies might also address the application of morphological analysis first, post-reconstructive surgery, and second, after biomechanical testing to better understand how much the testing equipment itself may affect tissue histomorphology.

## 5. Conclusions

This study’s comprehensive analysis of ATAAs in patients with BAV-A and TAV-A provides critical insights into the distinct structural and biomechanical characteristics associated with each valve type. The findings indicate that patients with BAV-A demonstrate significantly smaller AscAo maximal diameters, are younger, and are taller, with a higher prevalence of severe aortic valve stenosis compared to TAV-A patients. This suggests that BAV-A may confer an increased mechanical vulnerability, which is further supported by higher biomechanical strain and stress measurements observed in these patients. Moreover, the pronounced structural deterioration and increased fragmentation of collagen fibers in the BAV-A group underscore the more severe pathological alterations associated with this valve type. The presence of increased adipocyte accumulations in the TAV-A group and immune cell infiltration in the BAV-A group also highlights to complexity of the aneurysm condition. These distinct pathological features between BAV-A and TAV-A patients necessitate tailored clinical strategies for managing ATAAs. It is crucial for future research to focus on targeted therapies that address the specific structural and biomechanical vulnerabilities associated with each type of aortic valve aneurysm. By refining diagnostic and treatment approaches based on these insights, there is potential to significantly improve patient outcomes and reduce the morbidity associated with this serious condition.

## Figures and Tables

**Table 1:** Comparison of patients with ascending aortic aneurysms and bicuspid or tricuspid aortic valves: characteristics, laboratory indices, and diagnostic imaging.

Characteristic	Bicuspid Aortic Valve, *n* = 12	Tricuspid Aortic Valve, *n* = 14	*p*-Value
Age, years, median (IQR)	59.50 (51.25–66.25)	68.25 (62.25–74.00)	*p* **_MW_ = 0.0101**
Sex, male, ***n*** (%)	11 (91.7)	7 (50.0)	*p*_Chi_^2^_Y_ = 0.0617
Height, meters, mean (SD)	1.79 (0.10)	1.71 (0.09)	*p* **_St_ = 0.0382**
Weight, kg, median (IQR)	89.50 (80.50–96.00)	76.50 (66.75–103.5)	*p*_MW_ = 0.1632
BSA, m^2^, median (IQR)	2.06 (1.97–2.16)	1.89 (1.77–2.13)	*p*_MW_ = 0.0651
BMI, kg/m^2^, median (IQR)	27.72 (25.29–29.24)	26.94 (23.42–30.74)	*p*_MW_ = 0.7424
Hypertension, ***n*** (%)	7 (58.3)	12 (85.7)	*p*_Chi_^2^_Y_ = 0.2603
Diabetes mellitus, ***n*** (%)	1 (8.3)	0 (0)	*p*_Chi_^2^_Y_ = 0.9373
Significant coronary artery disease, ***n*** (%)	2 (16.7)	2 (14.3)	*p*_Chi_^2^_Y_ = 0.7059
Smoking, ***n*** (%)	4 (33.3)	6 (42.9)	*p*_Chi_^2^_Y_ = 0.9257
Arrhythmia, ***n*** (%)	3 (25)	5 (35.7)	*p*_Chi_^2^_Y_ = 0.8698
Severe aortic valve stenosis, ***n*** (%)	7 (58.3)	0 (0.0)	*p* **_Chi_^2^_Y_ = 0.0037**
Severe aortic valve regurgitation, ***n*** (%)	3 (25)	8 (57.1)	*p*_Chi_^2^_Y_ = 0.2092
EF of the left ventricle, %, median (IQR)	59.0 (55.8–60.0)	50.0 (48.3–63.3)	*p*_MW_ = 0.6755
Triglycerides, mmol/L, median (IQR)	1.31 (0.74–1.57)	1.00 (0.77–1.17)	*p*_MW_ = 0.1717
Total cholesterol, mmol/L, median (IQR)	4.10 (2.98–5.31)	4.52 (3.97–5.55)	*p*_MW_ = 0.2798
High density cholesterol, mmol/L, median (IQR)	1.11 (0.92–1.43)	1.38 (1.18–1.72)	*p* **_MW_ = 0.0194**
Low density cholesterol, mmol/L, median (IQR)	1.99 (1.58–3.80)	2.43 (2.29–3.51)	*p*_MW_ = 0.3953
AscAo maximum diameter, cm, median (IQR)	5.45 (5.33–5.70)	6.15 (5.60–6.75)	*p* **_MW_ = 0.0055**
AscAo length, cm, mean (SD)	12.23 (1.23)	11.71 (1.45)	*p*_St_ = 0.2523
Dilation of aortic root, ***n*** (%)	3 (25.0)	7 (50.0)	*p*_Chi_^2^_Y_ = 0.3671

Abbreviations: IQR—interquartile range; SD—standard deviation; BSA—body surface area; BMI—body mass index; EF—ejection fraction; AscAo—ascending aorta; *p*_St_—unpaired Student’s *t*-test; *p*_Chi2Y_—chi-square with Yates’ correction; *p*_MW_—Mann–Whitney U-test.

## Data Availability

Data are available from the corresponding author upon reasonable request, preferentially by e-mail: ivarsbrecs@gmail.com.
